# NR2F2 plays a major role in insulin-induced epithelial-mesenchymal transition in breast cancer cells

**DOI:** 10.1186/s12885-020-07107-6

**Published:** 2020-07-06

**Authors:** Baili Xia, Lijun Hou, Huan Kang, Wenhui Chang, Yi Liu, Yanli Zhang, Yi Ding

**Affiliations:** 1 Department of Pathophysiology, Weifang Medical University, Weifang, 261053 China; 2 Key Laboratory of Applied Pharmacology, Weifang Medical University, Weifang, 261053 China

**Keywords:** Breast cancer, NR2F2, Insulin, Epithelial-mesenchymal transition, Migration, Invasion metastasis

## Abstract

**Background:**

The failure of treatment for breast cancer usually results from distant metastasis in which the epithelial-mesenchymal transition (EMT) plays a critical role. Hyperinsulinemia, the hallmark of Type 2 diabetes mellitus (T2DM), has been regarded as a key risk factor for the progression of breast cancer. Nuclear receptor subfamily 2, group F, member 2 (NR2F2) has been implicated in the development of breast cancer, however its contribution to insulin-induced EMT in breast cancer remains unclear.

**Methods:**

Overexpression and knockdown of NR2F2 were used in two breast cancer cell lines, MCF-7 and MDA-MB-231 to investigate potential mechanisms by which NR2F2 leads to insulin-mediated EMT. To elucidate the effects of insulin and signaling events following NR2F2 overexpression and knockdown, Cells’ invasion and migration capacity and changes of NR2F2, E-cadherin, N-cadherin and vimentin were investigated by real-time RT-PCR and western blot.

**Results:**

Insulin stimulation of these cells increased NR2F2 expression levels and promoted cell invasion and migration accompanied by alterations in EMT-related molecular markers. Overexpression of NR2F2 and NR2F2 knockdown demonstrated that NR2F2 expression was positively correlated with cell invasion, migration and the expression of N-cadherin and vimentin. In contrast, NR2F2 had an inverse correlation with E-cadherin expression. In MDA-MB-231, both insulin-induced cell invasion and migration and EMT-related marker alteration were abolished by NR2F2 knockdown.

**Conclusions:**

These results suggest that NR2F2 plays a critical role in insulin-mediated breast cancer cell invasion, migration through its effect on EMT.

## Background

Breast cancer and diabetes are major worldwide health care problems. Accumulating evidence indicates that type 2 diabetes mellitus (T2DM) increases the risk of developing breast cancer [1, 2]. The potential molecular mechanisms contributing to this malignancy have been suggested by the role of hyperinsulinemia, a hallmark of T2DM, and a key risk factor for the development of breast cancer [3]. Long-term use of insulin analogs, insulin glargine, is associated with an increased risk of breast cancer in women with T2DM [4, 5]. Two large studies indicated that serum insulin is positively associated with breast cancer risk [6, 7]. Breast cancer has also been found associated with elevated levels of endogenous circulating insulin in non-diabetic patients [8]. A recent study illustrated that hyperinsulinemia promotes proliferation, migration and invasion of the human breast cancer cell line MDA-MB-231 by upregulation of urokinase plasminogen activator and its activation depends on production of reactive oxygen species (ROS) [9, 10]. Insulin promotes the growth of breast cancer cells in nude mice, and increases the proliferation and migration of MCF-7 human breast cancer cell line by upregulating insulin receptor substrate 1 and activating the Ras/Raf/ERK pathway [11, 12].

The nuclear receptor subfamily 2,group F, member 2 (NR2F2) gene encodes a member of the steroid thyroid hormone superfamily of nuclear receptors and is a member of chicken ovalbumin upstream promoter-transcription factors (COUP-TFs) superfamily, also known as COUP-TFII or ARP-1 [13–15]. It regulates glucose and lipid metabolism and is involved in the regulation of several important biological processes, such as neurogenesis, organogenesis, cellular differentiation during embryonic development and metabolic homeostasis [16]. Heterozygous NR2F2-knockout mice displayed lower basal level of insulin and enhanced insulin sensitivity compared to wild type mice [17, 18]. Recently, two opposing roles of NR2F2 on promoting or inhibiting tumorigenesis and metastasis have been reported. Higher expression of NR2F2 was associated with a worse prognosis and lymph node metastasis in human breast cancer cases [19]. In contrast, conditional ablation of NR2F2 severely compromised neoangiogenesis and suppressed tumor growth in xenograft mouse model [20]. Tumor growth and metastasis were inhibited in a spontaneous mammary-gland tumor model in the absence of NR2F2 by regulation of angiopoietin-1 [19]. Yet, the expression of NR2F2 has an inverse correlation with tumor grade in breast cancer [21]. Similarly, low NR2F2 transcription levels correlated with increased grade and lymph node metastasis based on the study of GEO and TCGA database [22] suggesting that high NR2F2 transcription levels are associated with favorable clinic outcomes through suppression of transforming growth factor - β (TGF-β) induced EMT.

The opposing effects of NR2F2 expression on cancer cell growth and metastasis indicate the complexity of its role in cancer. In the current study, we studied the effect of NR2F2 on insulin-mediated EMT in the breast cancer cells lines, MDA-MB-231 and MCF-7. We found that NR2F2 played a crucial role in insulin-mediated EMT in breast cancer cells by increasing vimentin and N-cadherin and inhibiting E-cadherin levels.

## Methods

### Cells culture and reagents

The human breast cancer cell lines MDA-MB-231 and MCF-7 were obtained from American Type Culture Collection (Manassas, VA, USA). MDA-MB-231 cells were cultured in RPMI1640 medium (Hyclone, LA, USA) with 10% fetal bovine serum (FBS, Gibco, NYC, USA) at 37 °C in 5% CO_2_ incubator. MCF-7 cells were grown in MEM medium (Hyclone, LA, USA) with 10% FBS and 0.01 mg/ml insulin (Sigma-Aldrich, SL, USA) at 37 °C in 5% CO_2_ incubator. Before insulin stimulation, cells were pre-seeded in plates with insulin-free medium for 24 h. Insulin was purchased from Sigma-Aldrich. Insulin was dissolved in HCI solution (pH 2.0). The final concentrations of HCI solution (pH 2.0) in the culture medium were less than 0.1%, and these amounts were also included in the corresponding controls.

### RNA isolation and quantitative real-time PCR (q-PCR)

Cells were seeded in 6-well plates at a density of 5 × 10^6^ cells/well in insulin-free medium for 24 h. The next day, cells were incubated in medium with or without insulin for certain time and then total RNA was extracted using RNeasy plus mini kit from Qiagen (Valencia, CA, USA) as previously described [23]. Total RNA (2 μg) was reverse-transcribed to cDNA (RT) with TaqMan Reverse Transcription Reagents. cDNA was amplified by PCR using the TaqMan probe for NR2F2 (Hs00819630_m1), E-cadherin (CDH1, Hs01023894_m1), N-cadherin (CDH2, Hs0098305_m1) and vimentin (Hs00958111_m1). Glyceraldehyde 3-phosphate dehydrogenase (GAPDH, Hs02758991_g1) was used as an endogenous control. Quantitative real-time PCR was performed using the TaqMan Universal PCR Master Mix. The PCR condition was denaturing at 95 °C for 15 s and annealing at 60 °C for 1 min with 40 cycles. Signals were analyzed by the Light Cycler480-II, Roche, USA. All probes and reagents for reverse-transcription and PCR were purchased from Thermo Fisher Scientific. Significant differences between the treatment and the control values were determined by a two-tailed Student’s *t* test with a nominal *P* value of < 0.05 considered significant.

### Protein extraction and Western blot

Cells (3 × 10^5^) were seeded into 6-well plate and incubated with or without insulin. As previously described [24], proteins were extracted using RIPA buffer containing protease inhibitor cocktail and PMSF 1 mM (Solarbio, PRC). After centrifugation (12,000 g for 15 min at 4 °C), the supernatants were collected for western blot analysis and the protein concentration was determined using BCA Protein Assay kit (CWBio, Beijing, China). Total protein (25 μg) was separated by 10% sodium dodecyl sulfate-polyacrylamide gel electrophoresis and transferred onto a polyvinylidene fluoride membrane (EMD Millipore, Billerica, MA, USA). The membranes were blocked with 5% non-fat dry milk in TBST for 1 h and then probed overnight at 4 °C with the following antibodies of NR2F2 (1:1000 dilution, PPMX, Tokyo, JP), β-actin (1:1000 dilution, Cell Signaling Technology, Boston, USA), vimentin (1:1000 dilution, Cell Signaling Technology, Boston, USA), N-cadherin (1:1000 dilution, Cell Signaling Technology, Boston, USA), and E-cadherin (1:1000 dilution, Cell Signaling Technology, Boston, USA). The membranes were then blotted with anti-mouse (1:5000 dilution, cat. no. A0216) and anti-rabbit (1:3500 dilution, cat. no. A0208) secondary antibodies (both from Beyotime Institute of Biotechnology, Shanghai, China) for 1 h at room temperature. The signal was detected using enhanced chemiluminescence (Immobilon Western Chemiluminescent Horseradish Peroxidase Substrate, EMD Millipore) and recorded on X-ray film. Results are expressed as percentage of control, mean ± S.D.

### RNA interference-mediated down regulation of NR2F2

The cells were seeded in 24-well plates at 30 to 50% confluence overnight and then changed medium to Opti-MEM® Reduced Serum Medium (Invitrogen, ThermoFisher, USA). 75 pmol of siRNA for human NR2F2 (Cat. no. 4390824, ID: s14021, Ambion, USA) was added into cells with Lipofectamine 3000 (Thermo Fisher Scientific, USA) for siRNA transfecton as descried before [23]. A non-target siRNA (Cat. no. 4390843, Ambion, USA) was used as a negative control. Eight hours later, the medium was changed back to regular medium. The mRNA and protein expression of NR2F2 was measured by quantitative RT-PCR and western blot separately to determine the transfection efficiency.

### Plasmid-mediated overexpression of NR2F2

The cells were seeded in 24-well plates at 80 to 90% confluence overnight and then changed medium to Opti-MEM® Reduced Serum Medium1 μg of plasmid for human NR2F2 (pCMV-MCS-IRES-EGFP-SV40-Neomycin, Genechem, Shanghai, China) was added to each well with Fugene HD (Promega,Madison, USA). An empty vector was used as negative control. Twenty-four hours later, the mRNA and protein expression of NR2F2 was measured by quantitative RT-PCR and western blot separately to confirm that NR2F2 was overexpressed successfully and then cells were treated for the following experiments.

### Cell migration assay

Cell migration was examined with wound-healing experiments. Culture cells were seeded in 24-well plates at a confluence of 80~90%. After 24 h, the confluent monolayer cells were scratched with a 200 μl micropipette tip, washed twice with PBS to get rid of the excess cells and treatment was applied. The cells were photographed and the distance of migration was measured under Leica Microsystems CMS GmbH (Leica, Germany).

### Cell invasion assay

Cells in suspension were plated at the density of 2 × 10^5^/ml (150 ul/well) into the matrigel-coated insert of a transwell chamber (Corning, PRC). The lower chamber was filled with 60 μl of medium containing 10% FBS to induce chemotaxis. Twenty-four hours later, the non-migrated cells in the upper chamber were gently scraped away by cotton swab, and adherent cells present on the lower surface of the insert were fixed with methanol, stained with 1% toluidine / 1% borax solution, six fields were randomly chosen from each chamber membrane and counted using Image J software under microscope (Leica Microsystems CMS GmbH).

### Cell proliferation and viability

Cells were seeded in 96-well plates at 4 × 10^3^ per well in growth medium complemented with 10% FBS. Cell proliferation/viability was evaluated using a [3- (4,5-dimethyl-2-thiazolyl)-2,5-diphenyl-2-H-tetrazolium bromide] (MTT, SIGMA, USA) assay at 6, 12, 24 and 48 h after treatment. Cells were incubated with MTT solution (5 mg/ml) in culture medium (20 μl per well) at 37 °C for 4 h. After centrifugation the medium was carefully removed, 100 μl isopropanol was added to each well to dissolve the formazan crystals. Optical density was recorded at 570 nm using a ultraviolet spectrophotometer (Bio-tek instruments, USA).

### Statistical analysis

A statistical analysis was performed using Student’s *t*-test for comparing between 2 observations results, and one-way ANOVA was used for multiple comparisons. The values are provided as the mean ± standard deviation (SD) and were considered statistically significant at *P* < 0.05. All experiments performed in vitro were biologically repeated at least three times unless otherwise indicated.

## Results

### Insulin promotes the migration and invasion of breast cancer cells

**Fig. 1:**
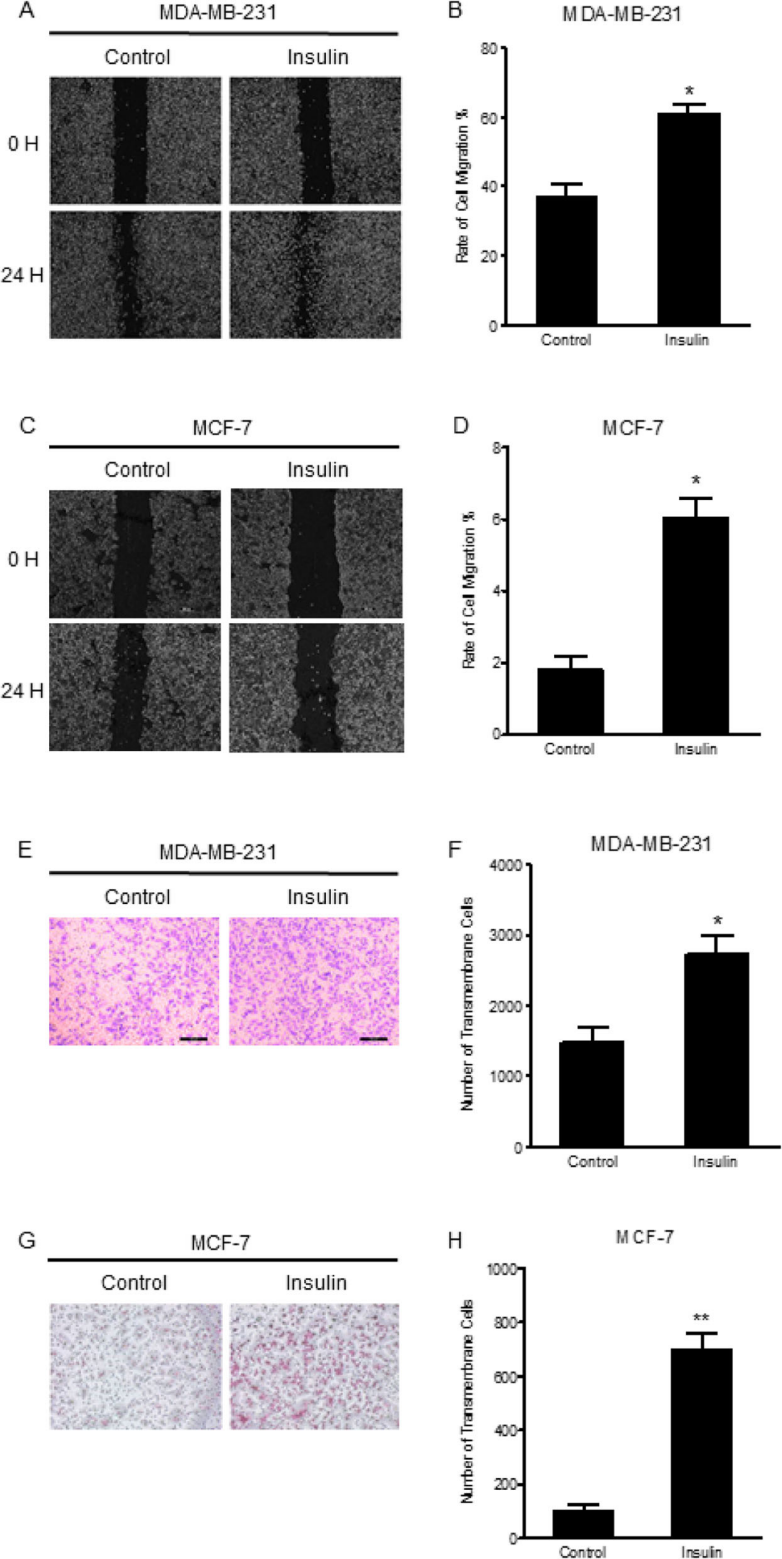
The effect of insulin on invasion and migration in MDA-MB-231 and MCF-7. **a** and **b** MDA-MB-231 cells and **c** and **d** MCF-7 cells were scratched by a micropipette tip and then incubated with or without insulin for 24 h (both *n* = 4). Cells were photographed and the distance was measured under microscope. **e** and **f** MDA-MB-231 cells and **g** and **h** MCF-7 cells were seeded separately into 8 μm-pole transwell chamber at 80~90% confluence. After starving for 12 h, monolayer cells were treated with or without insulin for 48 h, and then the filter was washed, stained and photographed under a microscope (both *n* = 4). *P < 0.05, ** *P* < 0.01 vs. control

The effect of insulin on cell migration and invasion was evaluated in a mesenchymal type (MDA-MB-231) and epithelial type (MCF-7) cancer cells. Following incubation with insulin (4 μM for 24 h), the rates of migration (Fig. 1a-d) and invasion (Fig. 1e-h) in both MDA-MB-231 and MCF-7 significantly increased. However, the rates of migration and invasion in the MDA-MB-231 cells were greater than that found in MCF-7 cells.

### Insulin enhances the migration and invasion of breast cancer cells through alterations in EMT-related molecular markers

**Fig. 2:**
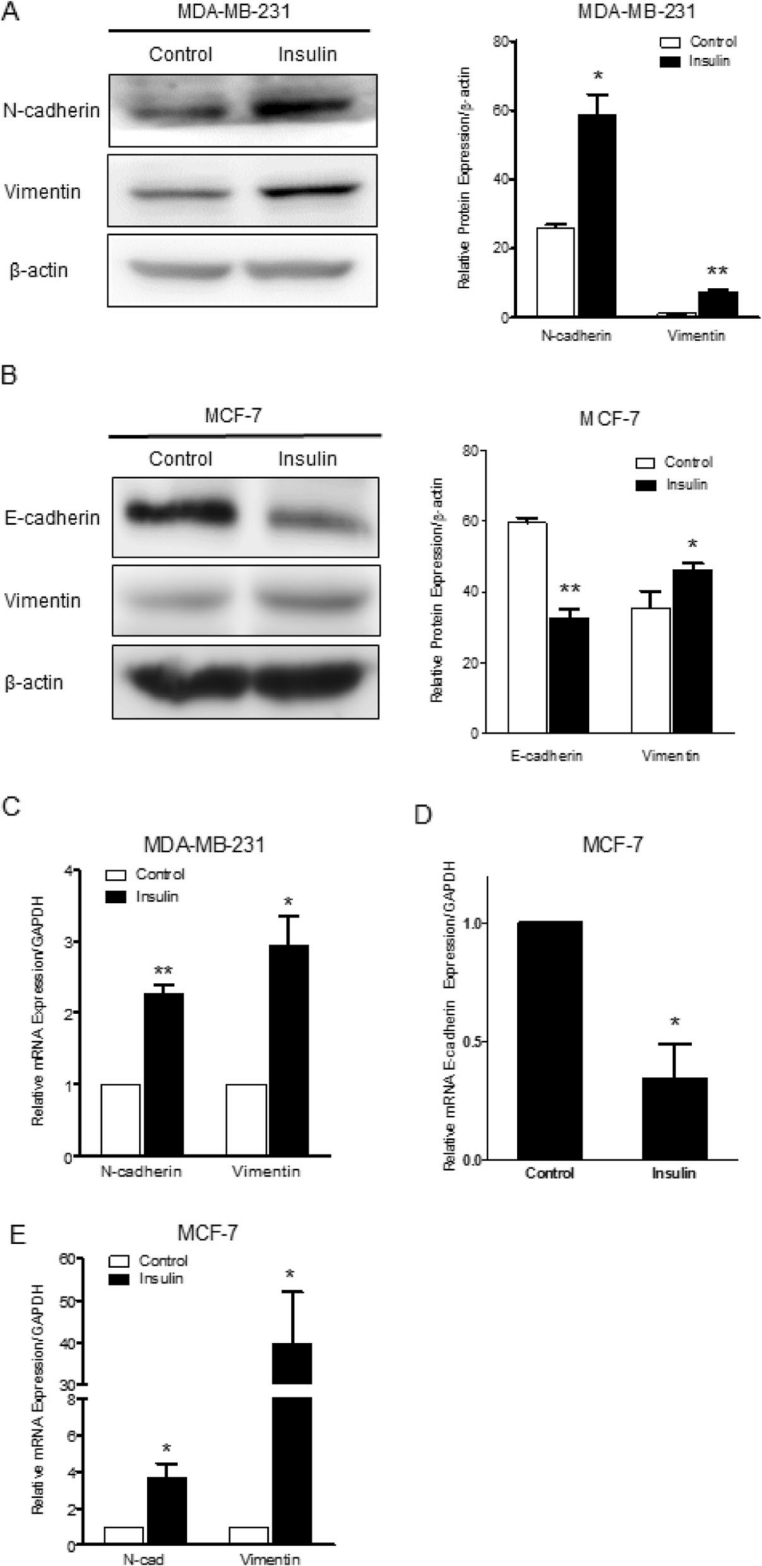
The effect of insulin on EMT marker expression. **a** MDA-MB-231 cells were treated with or without insulin (4 μM) for 24 h. The N-cadherin and vimentin proteins expression were detected by western blot (n = 4). **b** MCF-7 cells were treated with or without insulin (4 μM) for 12 h. The E-cadherin and vimentin protein expression were detected by western blot (*n* = 3). **c** MDA-MB-231 cells were treated with or without insulin (4 μM) for 24 h, N-cadherin and vimentin mRNAs expression were detected by real-time RT-PCR. **d** and **e** MCF-7 cells were treated with or without insulin (4 μM) for 24 h. The E-cadherin, N-cadherin and vimentin mRNAs expression were detected by real-time RT-PCR. * *P* < 0.05, ** *P* < 0.01 vs. control

In order to assess whether EMT was involved in their migration and invasion caused by insulin treatment, the expression of specific EMT molecular markers were determined. Under basal condition, MCF-7 cells expressed higher level of E-cadherin and less vimentin (N-cadherin was undetectable, data not shown) while MDA-MB-231 cells expressed higher level of N-cadherin and less vimentin (Fig. 2a-b). Following incubation with insulin (4 μM for 24 h) in MCF-7 cells, insulin down regulated E-cadherin mRNA expression and upregulated vimentin mRNA expression (Fig. 2d). Similarly, insulin induced N-cadherin and vimentin mRNA expression in MDA-MB-231 cells (Fig. 2c).

### Upregulation of NR2F2 expression by insulin

**Fig. 3:**
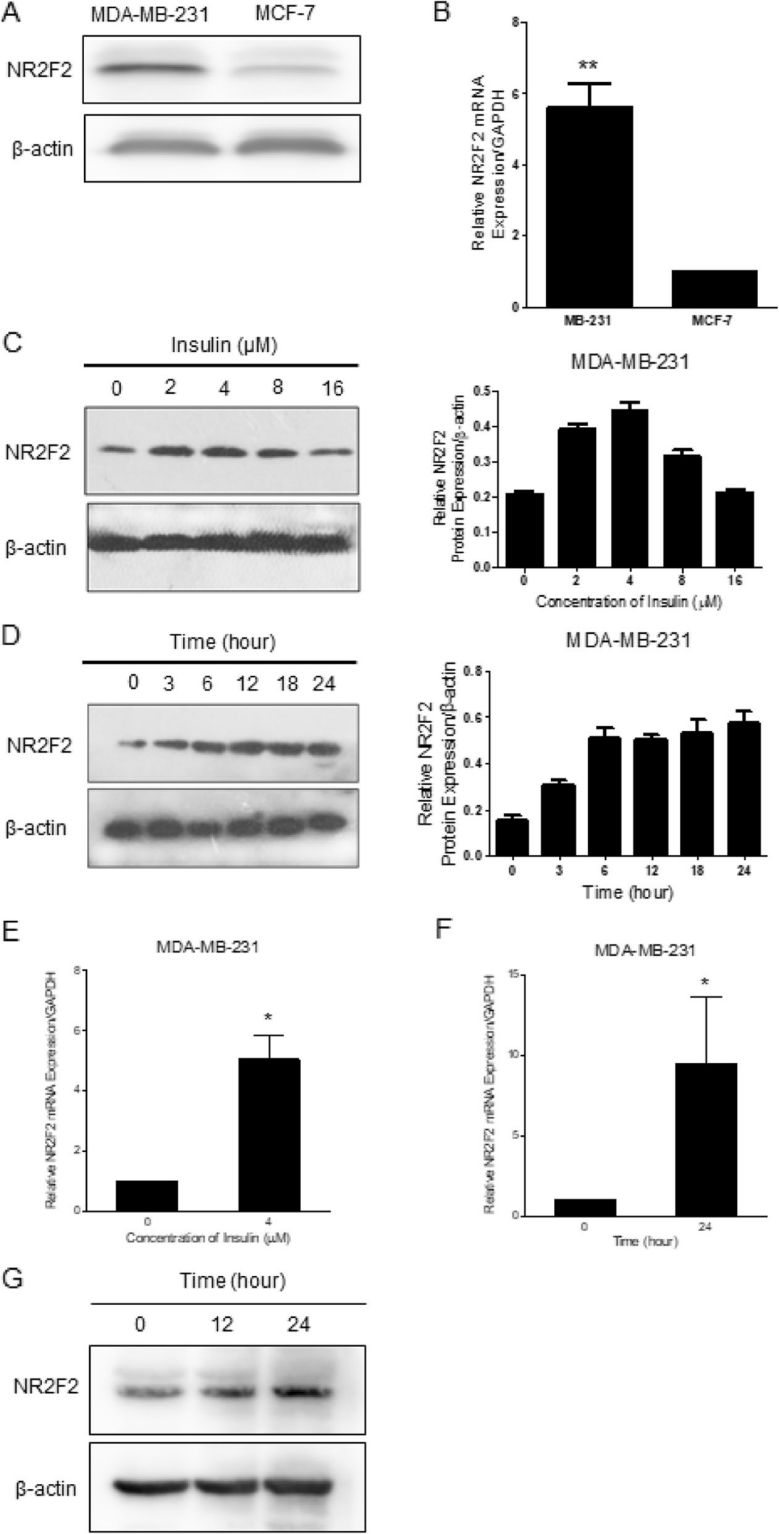
The effect of insulin on NR2F2 expression in breast cancer cells. **a** Protein expression of NR2F2 was measured in MDA-MB-231 and MCF-7 cells. **b** mRNA expression of NR2F2 was measured in MDA-MB-231 and MCF-7 (*n* = 5). **c** Dose effect of insulin at concentration of 0, 2, 4, 8, 16 μM for 24 h on NR2F2 protein expression in MDA-MB-231 cells. **d** Time effect of insulin at 4 μM for 0, 3, 6, 12, 24 h on NR2F2 protein expression in MDA-MB-231 cells. **e** NR2F2 mRNA expression was measured in MDA-MB-231 after insulin incubation for 24 h. **f** mRNA expression of NR2F2 in MCF-7 cells was detected after cultured in insulin for 12 and 24 h. **g** MCF-7 cells were cultured in insulin for 12 and 24 h, NR2F2 protein expression was detected. **P<0.01 vs. control

Basal expression of NR2F2 protein was greater in MDA-MB-231 cells than MCF-7 cells (Fig. 3a-b). In MDA-MB-231 cells, insulin treatment upregulated NR2F2 protein expression in a dose and time-dependent effect (Fig. 3c-d). In addition, after 24 h, insulin was associated with increased NR2F2 mRNA expression (Fig. 3e). Levels of NR2F2 mRNA and protein in MCF-7 cells were increased by insulin at 12 h and 24 h (Fig. 3f-g).

### NR2F2 over-expression enhanced EMT related markers and MCF-7 breast cancer cell invasion

**Fig. 4:**
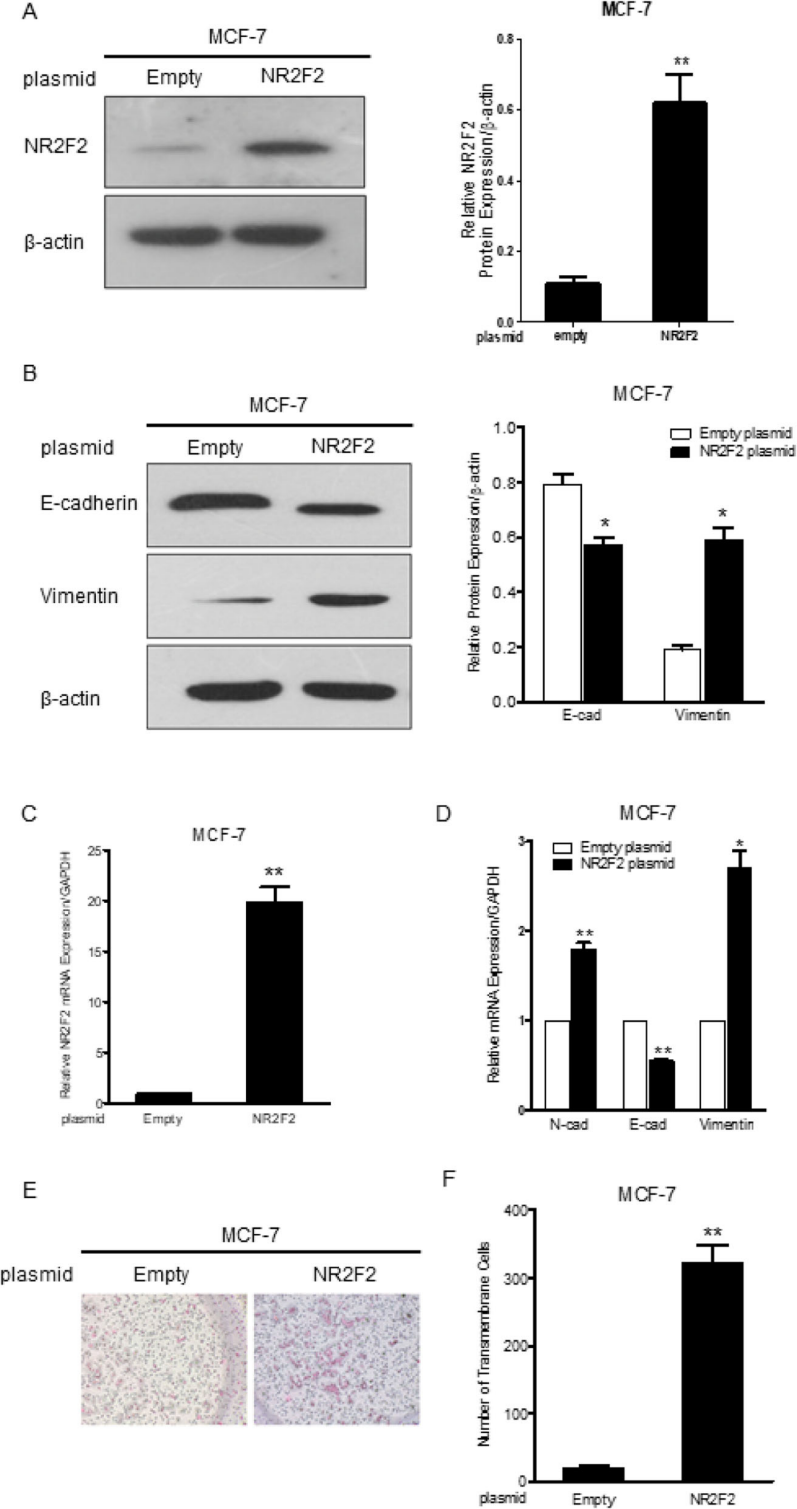
The effect of NR2F2 overexpression on EMT in MCF-7. **a** MCF-7 cells were transfected by NR2F2 overexpressed plasmid and empty vector, the protein expression of NR2F2 was detected by Western blot. **b** The NR2F2 mRNA expression was detected by real-time PCR. **c** The protein expression of EMT markers including E-cadherin and vimentin were detected by western blot in cells transfected with empty or NR2F2 overexpressed plasmid (*n *= 5). **d** The mRNA expression of EMT markers was detected by quantitive real-time PCR in cells transfected with empty or NR2F2 overexpressed plasmid (*n* = 5). **e** and **f** Plasmid transfected MCF-7 cells were seeded into the 8 μm-pole transwell chamber for 48 h. The number of transmembrane cells was counted (*n* = 4). *P < 0.05, **P < 0.01 vs. empty

In an attempt to find whether NR2F2 can affect EMT related protein expression, NR2F2-vector and empty vector plasmid DNA were transfected into MCF-7 cells respectively. In comparison to empty plasmid transfected cells, NR2F2 mRNA expression level in the NR2F2-plasmid DNA transfected cells increased up to 20 fold (Fig. 4b). MCF-7 cells had low basal levels of endogenous NR2F2 protein expression, however, following NR2F2 plasmid DNA transfection, the NR2F2 protein expression increased up to 4–6 fold (Fig. 4a). The expression levels of N-cadherin and vimentin mRNA were upregulated while the level of E-cadherin mRNA expression was downregulated by NR2F2 overexpression (Fig. 4d). The level of protein expression of E-cadherin was downregulated and vimentin upregulated by NR2F2 overexpression in MCF-7 cells (Fig. 4c). The transfected cells were evaluated by the cell invasion assay. The number of transmembrane cells significantly increased with NR2F2 overexpression in comparison to empty vector control (Fig. 4f-g). All these suggest that NR2F2 promotes EMT of breast cancer cells.

### NR2F2 knockdown attenuated insulin-induced EMT in MDA-MB-231 cells

**Fig. 5:**
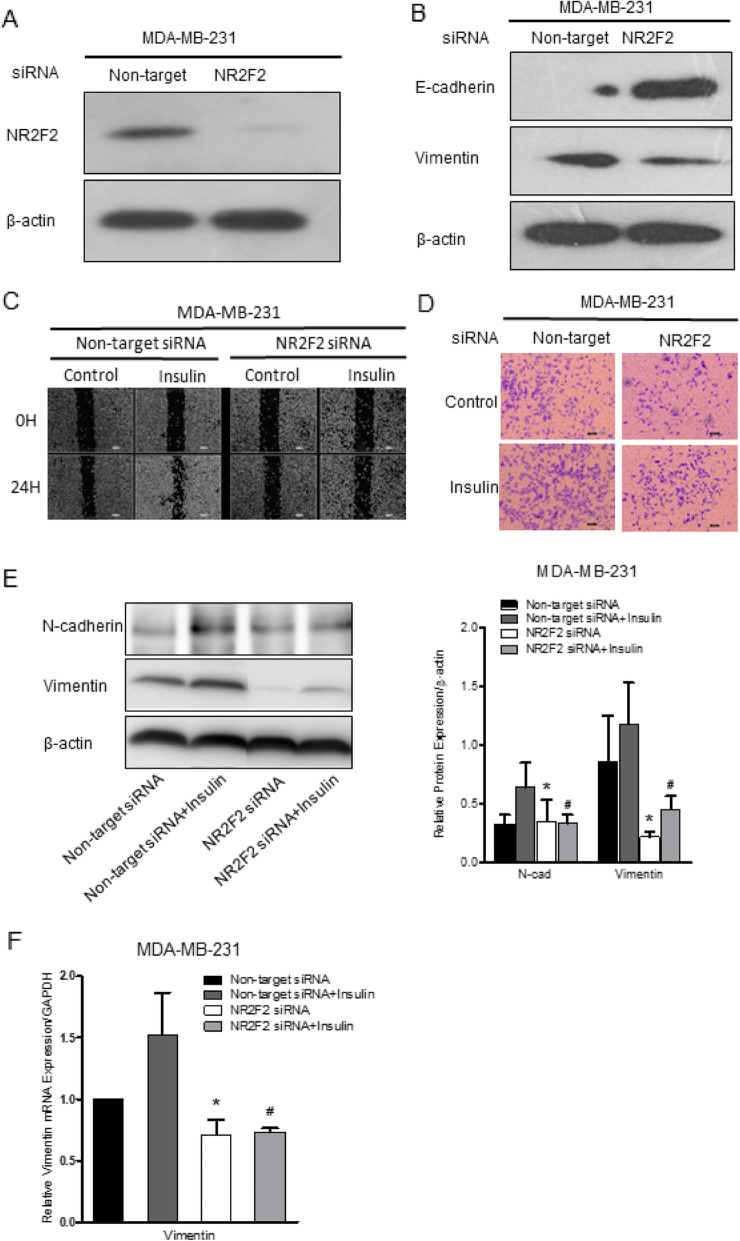
The effect of NR2F2 siRNA on EMT in MDA-MB-231. **a** MDA-MB-231 cells were transfected with NR2F2 siRNA and non-target siRNA. The protein expression of NR2F2 was measured by western blot. **b** The EMT marker E-cadherin and vimentin protein expression were examined by Western blot. **c** MDA-MB-231 cells were transfected with siRNA for 48 h, scratched by a micropipette tip and then cultured with or without insulin (4 μM) for 24 h. Cells were photographed and the distance was measured under microscope. **d** The invasion ability of siRNA transfected MDA-MB-231 cells induced by insulin was detected with transwell experiment. **e** Protein expressions of EMT markers were measured in siRNA transfected MDA-MB-231 cells under insulin treatment (*n* = 4). **f** After incubation with or without insulin, mRNA expression of EMT markers were measured in siRNA transfected MDA-MB-231 cells (*n* = 5). *P < 0.05 vs. non-target siRNA, ^#^*P* < 0.01 *vs.* non-target siRNA with insulin

MDA-MB-231 cells are characterized by high metastasis potential and with high expression of NR2F2. MDA-MB-231 cells were transfected with NR2F2 siRNA and non-target siRNA was used as a negative control. Forty-eight hours later, NR2F2 protein expression indicated that NR2F2 siRNA successfully reduced NR2F2 protein expression levels up to 90% (Fig. 5a). NR2F2 knockdown with siRNA treatment significantly inhibited the protein expression of vimentin and increased E-cadherin protein levels (Fig. 5b). Cell migration was also attenuated with NR2F2 silencing (Fig. 5c). Insulin-induced upregulation of N-cadherin and vimentin mRNA was inhibited by NR2F2 knockdown (Fig. 5e). Moreover, protein expression of N-cadherin and vimentin induced by insulin was parallel to mRNA expression (Fig. 5f). Thereafter, MDA-MB-231 cell migration and invasion was inhibited by NR2F2 silencing and insulin promotion (Fig. 5c-f). These results suggest that NR2F2 is an essential factor in the pathway of insulin-induced EMT.

## Discussion

We demonstrate that NR2F2 promotes EMT of breast cancer cell lines, resulting in enhanced breast cancer cells migration and invasion in vitro*.* Importantly, our NR2F2 knockdown experiments confirm the major role that NR2F2 plays in insulin-mediated EMT. These findings support the molecular mechanism by which hyperinsulinemia, which is common in obesity and T2DM, promotes breast cancer development and metastasis and thus contributes to poor outcomes in breast cancer.

Hyperinsulinemia is a hallmark of T2DM and has been recognized as a key risk factor for the initiation and progression of breast cancer [3, 25, 26]. Long-term use of insulin analogs, insulin glargine, is associated with an increased risk of breast cancer in women with T2DM [4, 5]. Further, insulin promotes breast cancer cell proliferation and migration [27]. A clinical investigation also indicated that fasting serum insulin is an independent risk factor for benign proliferative breast disease which is associated with early stages of breast cancer development [28]. Insulin induces downregulation of E-cadherin expression, accompanied with an increase in N-cadherin and vimentin expression in mammary non-tumorigenic epithelial cell line MCF10A [29] suggesting that insulin has a potential to mediate the EMT. EMT is a normal biological process of disaggregating structured epithelial units to enable cell movement and morphogenesis, especially involved in embryonic development, tissue remodeling and wound-healing [30–32]. Moreover, EMT has been implicated in migration and invasion of cancer cells and plays crucial roles in cancer progression and metastasis by the loss of cell polarity, degradation of basement membrane, dismantling of adherent junctions and desmosomes, and the acquisition of mesenchymal characteristics including loss of expression of cell adhesion molecules (such as E-cadherin) and increased expression of vimentin, N-cadherin, matrix metalloproteinase (MMP). Most of these proteins are regulated by specific transcription factors [33, 34].

Insulin and insulin-like growth factor promote EMT in gastric, colon, hepatic and breast cancer [35–39]. Hyperinsulinemia is a key risk factor for patients with breast cancer. Increased levels of fasting serum insulin is associated with distant tumor recurrence and death in women with early breast cancer and high levels of fasting insulin is an indicator of a poor prognosis in women with breast cancer [8]. In our current study, the effects of insulin on EMT in two different phenotype breast cancer cells lines, MDA-MB-231 and MCF-7 were assessed. We found that the migration and invasion of both types of cancer cells were increased by insulin treatment. The expression of epithelial characteristic marker, E-cadherin was downregulated while mesenchymal type markers of vimentin, N-cadherin were upregulated by insulin treatment. These findings are consistent with the report that insulin induces downregulation of E-cadherin expression, accompanied with an increase in N-cadherin and vimentin expression in epithelial cell line MCF10A [29]. Accumulating evidence indicated a hormone nuclear receptor NR2F2 which highly expressed in mesenchymal stromal (stem) cells and epithelial progenitors but not expressed in mature epithelial cells is related to EMT [40–42]. We sought to identify whether NR2F2 is a regulator of EMT in breast cancer cells and compared NR2F2 expression between MDA-MB-231 and MCF-7 cells. MDA-MB-231 with mesenchymal type cell character, expressed much higher level of NR2F2 than the epithelial phenotype, MCF-7 cells, implying that NR2F2 possibly played a role in EMT. Further, we overexpressed NR2F2 in MCF-7 cells and found that cell migration and invasion were enhanced significantly, E-cadherin expression level was downregulated, and vimentin level was upregulated. Thus, our finding that overexpression of NR2F2 promoted the EMT in MCF-7 cells, is consistent with other reports, which found that NR2F2 is highly expressed in an invasive colon cancer cell line HT29, compared with a normal colonic cell line CCD-18Co and that NR2F2 regulated the EMT through crosstalk with TGF-β signalling [43].

No prior publications were found describing the effects of insulin on the expression level of NR2F2 in cancer cells. We found that insulin upregulated NR2F2 expression in MCF-7 and MDA-MB-231 breast cancer cells. Insulin increased NR2F2 expression at the mRNA or protein level in a dose- and time-dependent manner. A recent study support these observations by noting that EGF-mediated MAPK signaling pathway significantly induced NR2F2 expression in human breast cancer cell lines [18]. Previous studies describe insulin activated critical intracellular signaling pathways including phosphatidylinositol 3-kinase/AKT kinase (PI3K/Akt) pathway or RAF kinase/mitogen activated protein kinase (Ras-MAPK pathway) to promote breast cancer growth and invasion [44]. It is also known that NR2F2 plays a critical role in regulating insulin secretion in specialized pancreas β cells and insulin inhibits NR2F2 expression in these pancreas β cells via Foxo1 [45, 46].

In order to identify that whether EMT induced by insulin in breast cancer cells is mediated by NR2F2, we performed loss of function experiments in our in vitro model. In this study, endogenous NR2F2 knockdown with siRNA in MDA-MB-231 cells attenuated their migration and invasion, and the expression of E-cadherin in these cells increased while N-cadherin and vimentin decreased. This further confirmed the effect of NR2F2 on EMT in breast cancer cells. Notably, NR2F2 silencing mitigated insulin induced N-cadherin and vimentin expression at mRNA and protein level. Insulin mediated EMT was abolished when NR2F2 was knockdown in MDA-MB-231 cells, implying that NR2F2 mediates the effects of insulin on EMT, however the mechanism by which NR2F2 increases the N-cadherin, vimentin and inhibits E-cadherin remains unclear.

Our results suggest that NR2F2 may play an important role in breast cancer metastasis and thus may be a potential drug target to prevent metastasis in breast cancer with hyperinsulinemia patients or breast cancer with type 2 diabetes patients. Although NR2F2 is essential for tissue differentiation and cardiovascular development during embryonic stage, recent studies have showed that NR2F2 has little impact on the normal adult physiological function [20]. Although the physiological ligand of NR2F2 has not yet been identified in human body, 4-methoxynaphthol as an inhibitor of NR2F2 has been found with a yeast one-hybrid assay and validated in mammalian cells [47].

## Conclusions

Our results suggest that insulin promotes breast cancer cell invasion, migration by upregulating expression of NR2F2, which plays a critical role in insulin-mediated breast cancer cell invasion, migration through its effect on EMT.

## Data Availability

All data generated or analyzed during this study are included in this published article.

## Abbreviations

NR2F2: Nuclear receptor subfamily 2, group F, member 2
EMT: Epithelial-mesenchymal transition
T2DM: Type 2 diabetes mellitus
COUP-TFs: Chicken ovalbumin upstream promoter transcription factors
